# Probing expression of E-selectin using CRISPR-Cas9-mediated tagging with HiBiT in human endothelial cells

**DOI:** 10.1016/j.isci.2023.107232

**Published:** 2023-06-30

**Authors:** Lydia Ogrodzinski, Simon Platt, Joelle Goulding, Cameron Alexander, Tracy D. Farr, Jeanette Woolard, Stephen J. Hill, Laura E. Kilpatrick

**Affiliations:** 1 Division of Physiology, Pharmacology and Neuroscience, School of Life Sciences, University of Nottingham, NG7 2UH Nottingham, UK; 2 Centre of Membrane Proteins and Receptors, University of Birmingham and Nottingham, The Midlands, Nottingham, UK; 3 Division of Molecular Therapeutics and Formulation, School of Pharmacy, Boots Building, University of Nottingham, NG7 2RD Nottingham, UK; 4 Division of Bimolecular Science and Medicinal Chemistry, School of Pharmacy, Biodiscovery Institute, University of Nottingham, NG7 2RD Nottingham, UK

**Keywords:** Molecular biology, Biotechnology, Cell biology

## Abstract

E-selectin is expressed on endothelial cells in response to inflammatory cytokines and mediates leukocyte rolling and extravasation. However, studies have been hampered by lack of experimental approaches to monitor expression in real time in living cells. Here, NanoLuc Binary Technology (NanoBiT) in conjunction with CRISPR-Cas9 genome editing was used to tag endogenous E-selectin in human umbilical vein endothelial cells (HUVECs) with the 11 amino acid nanoluciferase fragment HiBiT. Addition of the membrane-impermeable complementary fragment LgBiT allowed detection of cell surface expression. This allowed the effect of inflammatory mediators on E-selectin expression to be monitored in real time in living endothelial cells. NanoBiT combined with CRISPR-Cas9 gene editing allows sensitive monitoring of real-time changes in cell surface expression of E-selectin and offers a powerful tool for future drug discovery efforts aimed at this important inflammatory protein.

## Highlights

• E-selectin is expressed on endothelial cells in response to inflammatory cytokines
• CRISPR-Cas9 editing has added HiBiT to the N terminus of E-selectin in HUVECs
• Addition of purified LgBiT allows re-complementation of the nanoluciferase enzyme
• Real-time changes in cell surface expression of E-selectin have then be monitored

## Graphical abstract

**Figure:**
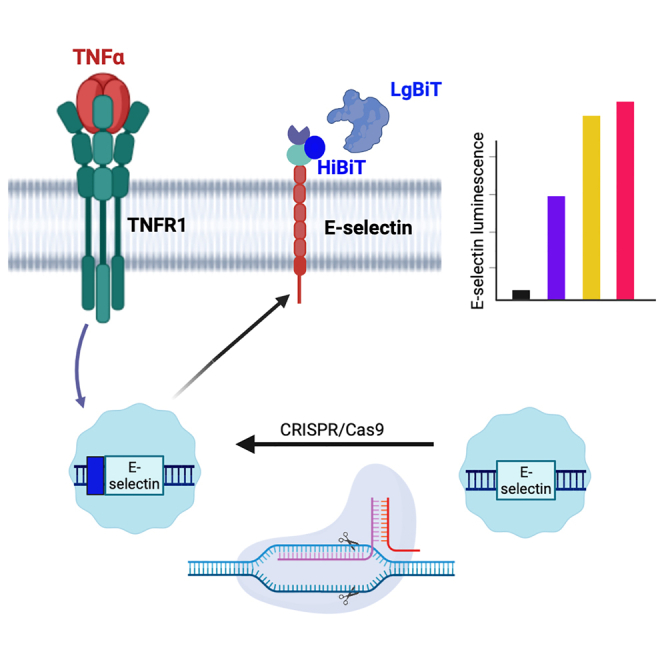

## Introduction

Selectins are a family of Ca^2+^-dependent C-type lectins present on the surface of numerous cell types in the cardiovascular system including endothelial cells (E- and P-selectin), platelets (P-selectin), and leukocytes (L-selectin).^1^ They interact with cell surface glycans to promote adhesion of hematopoietic cells to vascular surfaces and promote rolling of circulating leukocytes and their delivery to sites of inflammation.^2^^,^^3^^,^^4^ E-selectin (CD62E) is a 115 kDa adhesion molecule that is expressed exclusively by vascular endothelial cells.^2^ It is a single-chain transmembrane glycoprotein consisting of an N-terminal calcium-dependent (C-type) lectin domain, an epidermal growth factor domain, a chain of six consensus repeats, a transmembrane domain, and an intracellular cytoplasmic tail.^2^^,^^5^ Although E-selectin is constitutively expressed on the surfaces of endothelial cells of bone marrow and skin microvessels^2^^,^^6^^,^^7^ in most tissues, E-selectin expression is induced in response to inflammatory cytokines such as tumor necrosis factor alpha (TNFα), interleukin-1β (IL-1β), or lipopolysaccharide (LPS).^8^^,^^9^ E-selectin expression has also been associated with tumor angiogenesis and metastasis in a variety of cancers.^10^^,^^11^^,^^12^

E-selectin binds to the tetrasaccharide sialyl Lewis X (SLe^x^) structure.^12^ Ligands for E-selectin which possess sLe^x^-motifs include P-selectin glycoprotein ligand-1, CD44, and E-selectin ligand-1.^4^^,^^13^ In addition, a peptide that contains the sequence DITWDQLWDLMK that can bind E-selectin with high affinity has been discovered (E-selectin-binding peptide) using phage display.^12^^,^^14^ This peptide sequence has been used to target N-(2-hydroxypropyl)-methacrylamide-based co-polymer-doxorubicin conjugates to the tumor vasculature.^12^^,^^15^ Despite the well-established changes in E-selectin expression deduced by immunohistochemistry in vascular endothelial cells in response to inflammatory mediators, the detailed pharmacological characteristics of E-selectin expression in living endothelial cells have been more difficult to study. This is largely because of the lack of reagents to interrogate the properties of this C-type lectin in live cells in real time, and the limitations that are inherent in its normal low expression in endothelial cells in the absence of inflammation.

To investigate this further, we have used nanoluciferase^16^ and NanoLuc Binary Technology (NanoBiT)^17^^,^^18^^,^^19^ to investigate ligand-induced changes in the cell surface expression of E-selectin. To ensure that ligand-induced changes in cell surface expression were monitored in physiologically relevant cells under the control of their native promoters, we have used CRISPR-Cas9 genome editing to attach the 11 amino acid high-affinity NanoBiT tag HiBiT^17^^,^^19^ to the N terminus of endogenous E-selectin expressed in human umbilical endothelial cells (HUVECs). Following addition of exogenous purified 18-kDa membrane-impermeable LgBiT to HUVECs, it self-complements with HiBiT to reinstate the full-length nanoluciferase enzyme with subsequent luminescence identifying tagged proteins at the surface of living cells.^17^^,^^18^^,^^19^ Using this approach, we demonstrated ligand-induced expression of HiBiT-tagged E-selectin in primary HUVECs in response to TNFα, IL-1α, IL1-β, LPS, vascular endothelial growth factor (VEGF), and histamine.

## Results

### E-selectin expression in response to TNFα in fixed HUVECs

**Figure 1:**
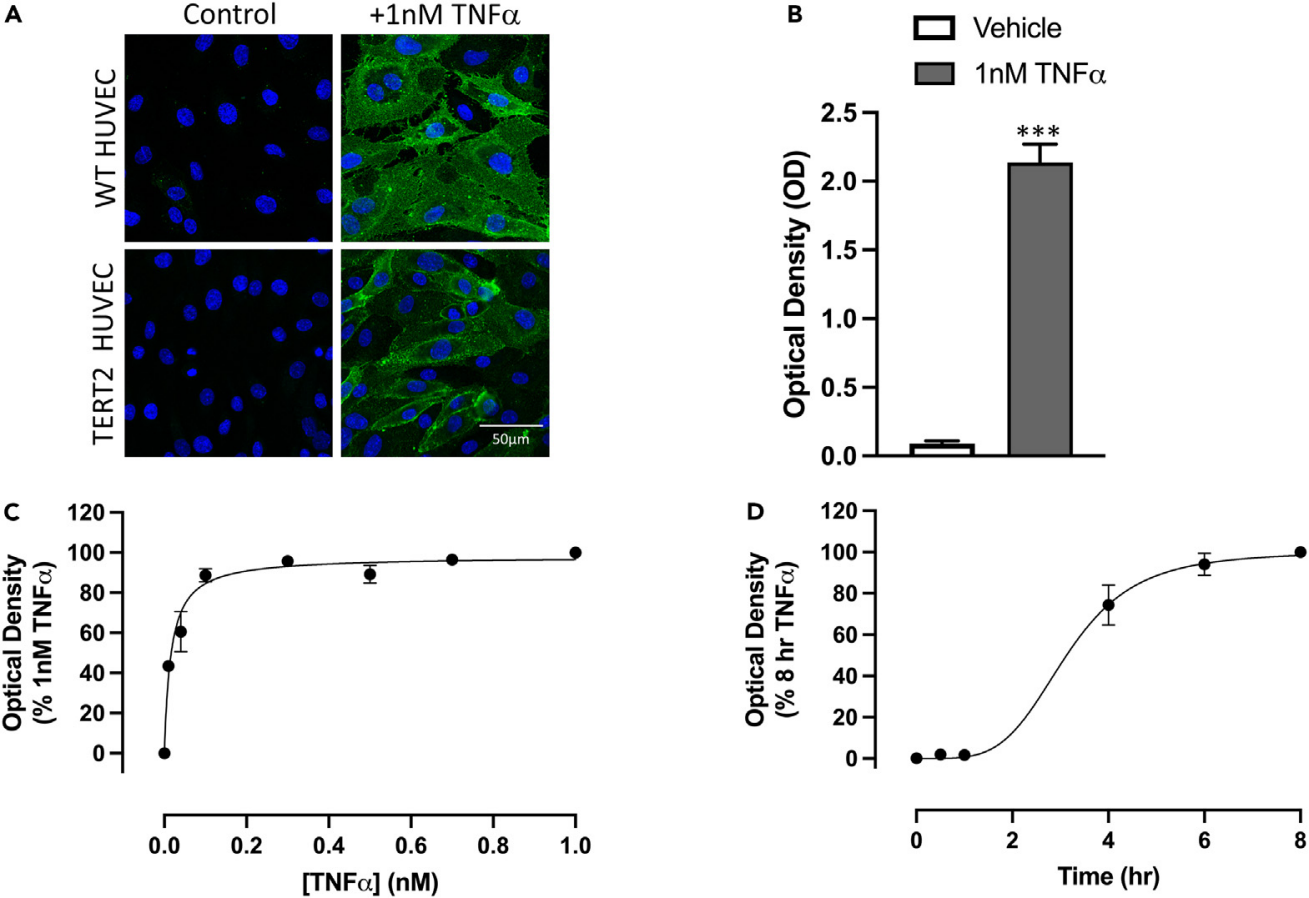
TNFα-stimulated increase in E-selectin expression in un-transfected HUVECs (A) Confocal images of TNFα-stimulated E-selectin expression in wild-type (WT) HUVECs and immortalized TERT2-HUVECs. Cells were stimulated with 1 nM TNFα for 6 (WT HUVECs) or 8 h (TERT2-HUVECs). H33342-stained nuclei are shown in blue and Alexa Fluor 488-labeled E-selectin in green. Scale bar represents 50 μm. (B–D) Quantification of E-selectin expression in WT HUVECs using an alkaline phosphatase secondary antibody and *p*-nitrophenol phosphate substrate. Values show mean ± S.E.M. of the optical densities obtained in five separate experiments in response to 1 nM TNFα. (C and D) Concentration-response curve (C) and time course (D) for TNFα-stimulated E-selectin expression in WT HUVECs. Values are mean ± S.E.M. of five separate experiments each performed in triplicate. In (C), values have been normalized to the response obtained with 1 nM TNFα. In (D), 1 nM TNFα was used, and values have been normalized to the response obtained at 8 h. ∗∗p < 0.001 compared to vehicle control (paired t test).

Initial experiments were undertaken in wild-type HUVECs to confirm that E-selectin was induced by TNFα over 6 h. Immunofluorescence detection of E-selectin in fixed cells using a monoclonal anti-E-selectin antibody and an Alexa Fluor 488-conjugated secondary antibody confirmed that robust E-selectin was observed in most cells following 6 h stimulation with 1 nM TNFα (Figure 1A; representative image of n = 5). Similar data were obtained in telomerase reverse transcriptase (TERT2)-immortalized HUVECs (Figure 1A). The response to TNFα in HUVECs was further characterized in fixed cells using an alkaline phosphatase-conjugated secondary antibody and its substrate *p*-nitrophenol phosphate to allow quantification of E-selectin expression as a color change that could be read on an absorbance plate reader (Figure 1B). These data showed that 1 nM TNFα produced a significant increase (p < 0.001) in optical density following 6 h incubation (Figure 1B). The response was concentration dependent and yielded an EC_50_ of 0.016 ± 0.003 nM (n = 5) (Figure 1C). Time course studies showed that the response to 1 nM TNFα peaked between 6 and 8 h and required more than 1 h stimulation to induce a significant change in expression (Figure 1D).

### E-selectin expression in CRISPR-Cas9 genome-edited HUVECs

**Figure 2:**
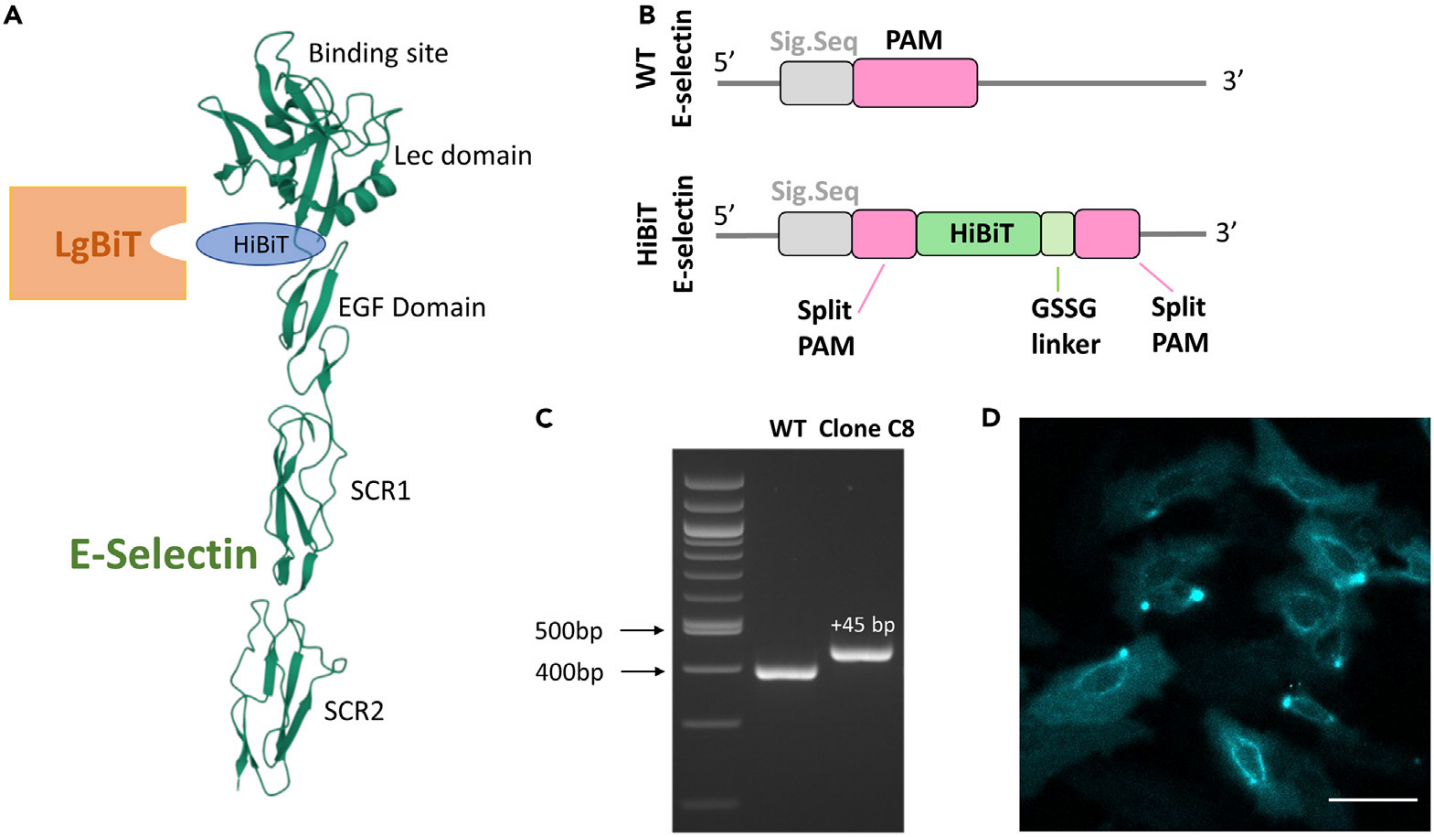
Genome editing of human E-selectin to attach the 11 amino acid HiBiT sequence to the N terminus (A) Schematic of the position of HiBiT on the N terminus of E-selectin and its re-complementation with LgBiT. The E-selectin structure shown is the extended structure of the N-terminal regions of E-selectin (PDB: 4C16) showing the binding site for glycomimetic 1/sLe^x^ and the N-terminal Lectin (Lec), EGF-like and first two SCR domains. (B) General genome-editing strategy showing the PAM sequence adjacent to the signal sequence of E-selectin and the insertion of HiBiT and a GSSG linker immediately following the signal sequence in the HiBiT-tagged E-selectin sequence. The editing disrupts the PAM sequence thus preventing any further modification by Cas9. (C) Agarose gel (3%) of a PCR amplified 370 bp fragment of the N-terminal region of E-selectin in wild-type HUVECs and HiBiT-edited TERT2-HUVEC clone C8. Successful homozygous edit of HiBiT TERT2-HUVEC clone C8 was demonstrated by the appearance of a single band representing the 45 bp addition (HiBiT + GSSG linker) in length of the PCR fragment compared to unedited HUVECs. A 100 bp ladder is also shown for comparison. (D) Bioluminescence imaging of HiBiT TERT2-HUVEC clone C8 cells expressing HiBiT-E-selectin under endogenous promotion. Cells were treated with 1 nM TNFα for 6 h and then incubated with purified LgBiT protein (50 nM in 0.1% BSA HBSS) for 20 min (37°C). Furimazine was then added (1:400 in 0.1% BSA HBSS) (5 min, RT) and 512 x 512 images were captured using bioluminescence filters (exposure 20 s, gain 200) on the Olympus LuminoView 200. Scale bar represents 50 μm. The image shown is representative of 5 similar experiments.

**Figure 3:**
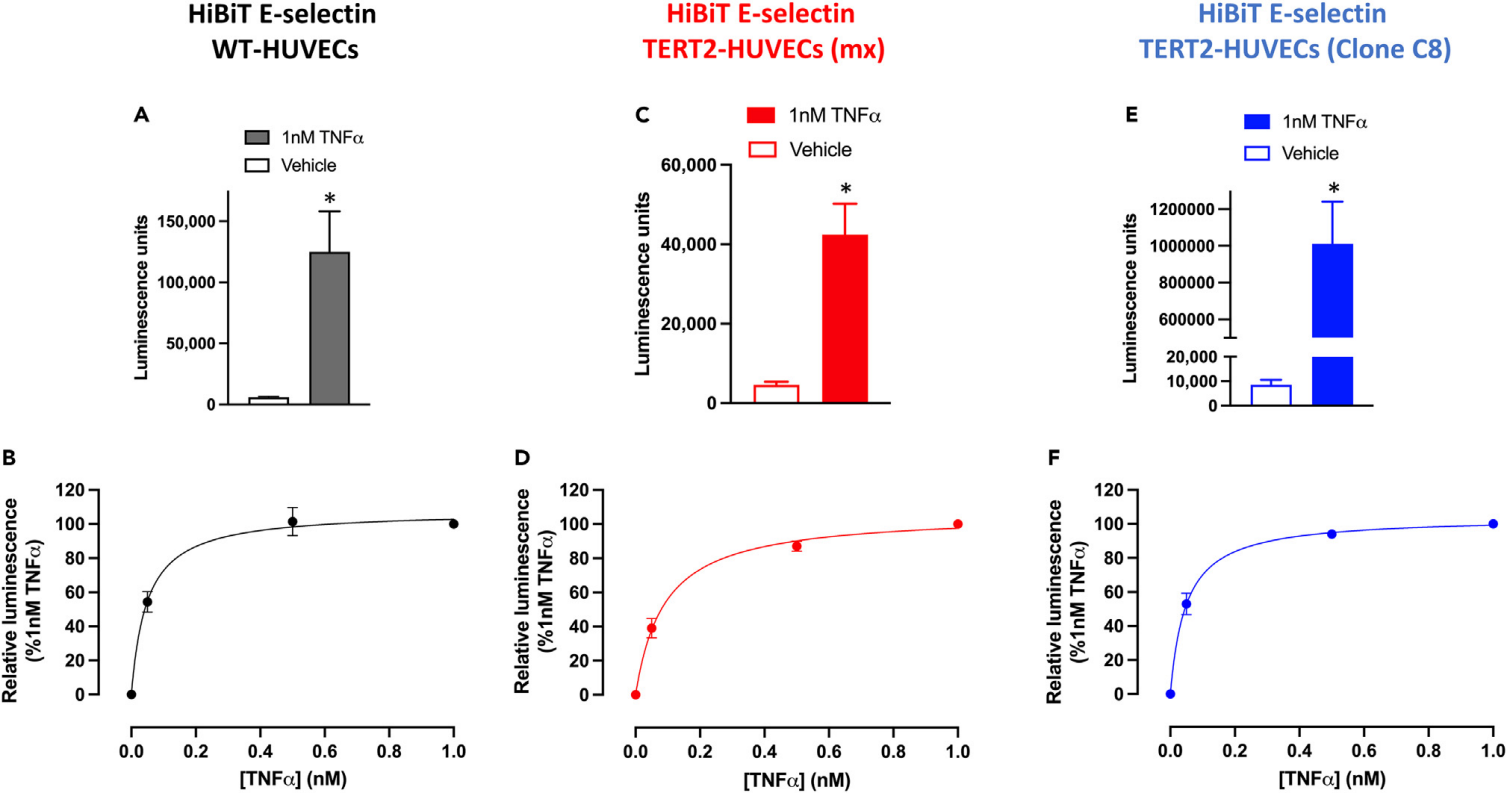
TNFα-stimulated nanoluciferase luminescence in HiBiT-E-selectin-edited HUVECs and HiBiT-edited TERT2-HUVECs (A, C, and E) Increase in luminescence (relative light units) under basal conditions and in response to 6 h stimulation with 1 nM TNFα in HiBiT genome-edited HUVECs (A), mixed populations of HiBiT-edited TERT2-HUVECs (C) and HiBiT TERT2 clone C8 HUVECs (E). (B, D, and F) Concentration-response curves for TNFα-stimulated luminescence in HiBiT HUVECs (B), HiBiT TERT2-HUVEC mixed populations (D), and HiBiT TERT2 clone C8 HUVECs (F). NanoBiT complementation was achieved by addition of purified LgBiT (50 nM) and furimazine substrate (1:400), and the plates were read using an BMG PHERAstar FS plate reader (450 nm, 30 nm bandpass). Values are mean ± S.E.M of 5 replicate experiments each performed in triplicate. ∗p < 0.05 compared to vehicle control (paired t test).

In order to evaluate E-selectin expression in living HUVECs, we undertook genome editing to introduce an 11 amino acid HiBiT tag onto the N terminus of endogenous human E-selectin in HUVECs. Initially, a 370 bp fragment of the N-terminal region of E-selectin was generated by PCR amplification and sequenced to confirm the lack of single-nucleotide polymorphisms in the target PAM site. A PAM site immediately following the signal sequence of E-selectin was then identified and used for CRISPR-Cas9 genome editing to introduce a HiBiT tag at the start of the coding sequence for E-selectin (Figure 2). Initial experiments with 1 nM TNFα stimulation of wild-type HiBiT-tagged HUVECs confirmed that this cytokine produced a robust stimulation of cell surface E-selectin expression (following re-complementation of HiBiT with exogenous membrane-impermeable purified LgBiT; Figure 3A) that was concentration dependent and yielded an EC_50_ value of 0.057 ± 0.011 nM (n = 5; Figure 3B).

However, one limitation of wild-type HUVECs is the limited number of passages (usually <9 passages) that they can be used over which they maintain their endothelial cell phenotype. As a consequence, the same genome-editing strategy was also undertaken in immortalized TERT2-HUVECs that do not have such strict passage limitations. These cells have been transfected with human TERT to prevent the normal reduction in telomerase expression and subsequent shortening of telomeres that leads to cell senescence.^20^ Mixed populations of TERT2-HUVECs expressing HiBiT-tagged E-selectin (TERT2 (mx) HUVECs) demonstrated a robust response to 1 nM TNFα that yielded an EC_50_ of 0.10 ± 0.03 nM (Figures 3C and 3D; n = 5) that was very similar to that observed in mixed populations of edited wild-type HUVECs. Both wild-type and TERT2-HUVECs retained their endothelial phenotype (as judged by expression of the endothelial marker CD31) in the presence and absence of genome editing (Figure S1). Mixed populations of TERT2-HiBiT-HUVECs were then subjected to dilution cloning to identify cells that were homozygous for HiBiT-E-selectin insertion.

Several clonal lines were identified with clones B4, H3, and C8 producing strong luminescence responses following TNFα stimulation (Figure S2). B4 and H3 were heterozygous while clone C8 was homozygous for HiBiT-E-selectin. Figure S3 shows the sequencing for clone C8 in the region of the HiBiT insertion. Short tandem repeat genetic profiles of wild-type and HiBiT-E-selectin gene-edited TERT2-HUVECs (clone C8) confirmed identical genome profiles for the TERT2-HUVEC and TERT2-HUVEC-HiBiT-E-selectin clone C8 cell lines (Table S1). Both cell lines had 86.7% identity with the genome profile of the original TERT2-HUVEC cell line deposited with ATCC (CRC-4053). This is above the 80% threshold generally accepted for declaring a match when accounting for genetic drift.^21^

**Figure 4:**
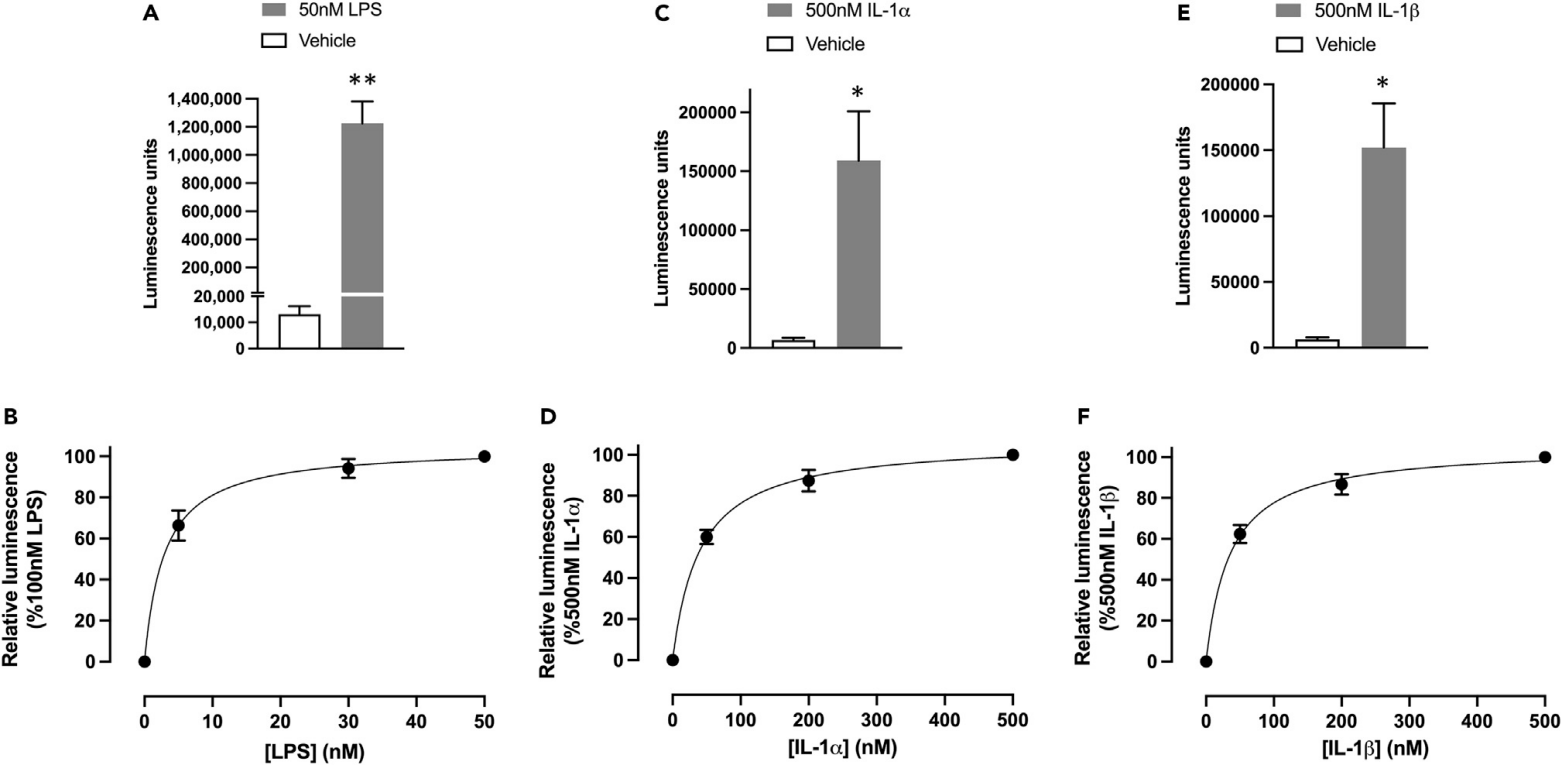
Cytokine-stimulated nanoluciferase luminescence in clonal HiBiT-edited E-selectin TERT2-HUVECs (A, C, and E) Increase in luminescence (relative light units) under basal conditions and in response to 6 h stimulation with 50 nM LPS (A), 500 nM IL-1α (C), and 500 nM IL-1β (E) in clone C8 HiBiT genome-edited TERT2-HUVECs. (B, D, and F) Concentration-response curves for cytokine-stimulated luminescence in clone C8 HiBiT TERT2-HUVECs in response to LPS (B), IL-1α (D), and IL-1β (F). NanoBiT complementation was achieved by addition of purified LgBiT (50 nM) and furimazine substrate (1:400), and the plates were read using a BMG PHERAstar FS plate reader (450 nm, 30 nm bandpass). Values are mean ± S.E.M of 5 replicate experiments each performed in triplicate. ∗p < 0.05 or ∗∗p < 0.01 compared to vehicle control (paired t test).

The homozygous clone C8 was selected for further study and produced a large concentration-dependent increase in HiBiT-E-selectin expression following 6 h stimulation with TNFα (Figures 3E and 3F). The EC_50_ value obtained for TNFα in this clonal line was 0.06 ± 0.02 nM (Figure 3F; n = 5). Analysis of the HiBiT insert in the genome sequence of this clone confirmed that it was homozygous for HiBiT insertion and yielded a single 45 bp increase in the size of the N-terminal region (Figure 2C). In contrast, other clones showed two bands equivalent to untagged E-selectin and HiBiT-tagged E-selectin consistent with heterozygous editing of the HiBiT tag on a single allele. In clone C8, the luminescence obtained following stimulation with 1 nM TNFα was sufficiently bright to image using bioluminescence imaging (Figure 2D). Significant increases in HiBiT E-selectin cell surface expression were also obtained in clone C8 in response to LPS, IL-1α, and IL-1β (Figure 4 and Table 1).

**Table 1:** EC_50_ values for cytokine-stimulated HiBiT E-selectin expression in gene-edited wild-type and TERT2-HUVECs

	WT-HUVECs EC_50_, nM (n)	Clone C8 TERT2-HUVECs EC_50_ nM (n)
TNFα	0.06 ± 0.01 (5)	0.06 ± 0.02 (5)
LPS	10.00 ± 2.86 (5)	2.80 ± 0.70 (5)
IL-1α	41.04 ± 18.08 (5)	32.84 ± 5.73 (5)
IL-1β	48.26 ± 15.47 (5)	30.90 ± 6.23 (5)

Values represent mean ± S.E.M. of five independent experiments. In each experiment, triplicate determinations were made. WT-HUVECs are mixed populations of gene-edited (HiBiT-tagged E-selectin) HUVECs while the TERT2-HUVECs represent a clonal cell line homozygous for HiBiT-tagged E-selectin.

**Figure 5:**
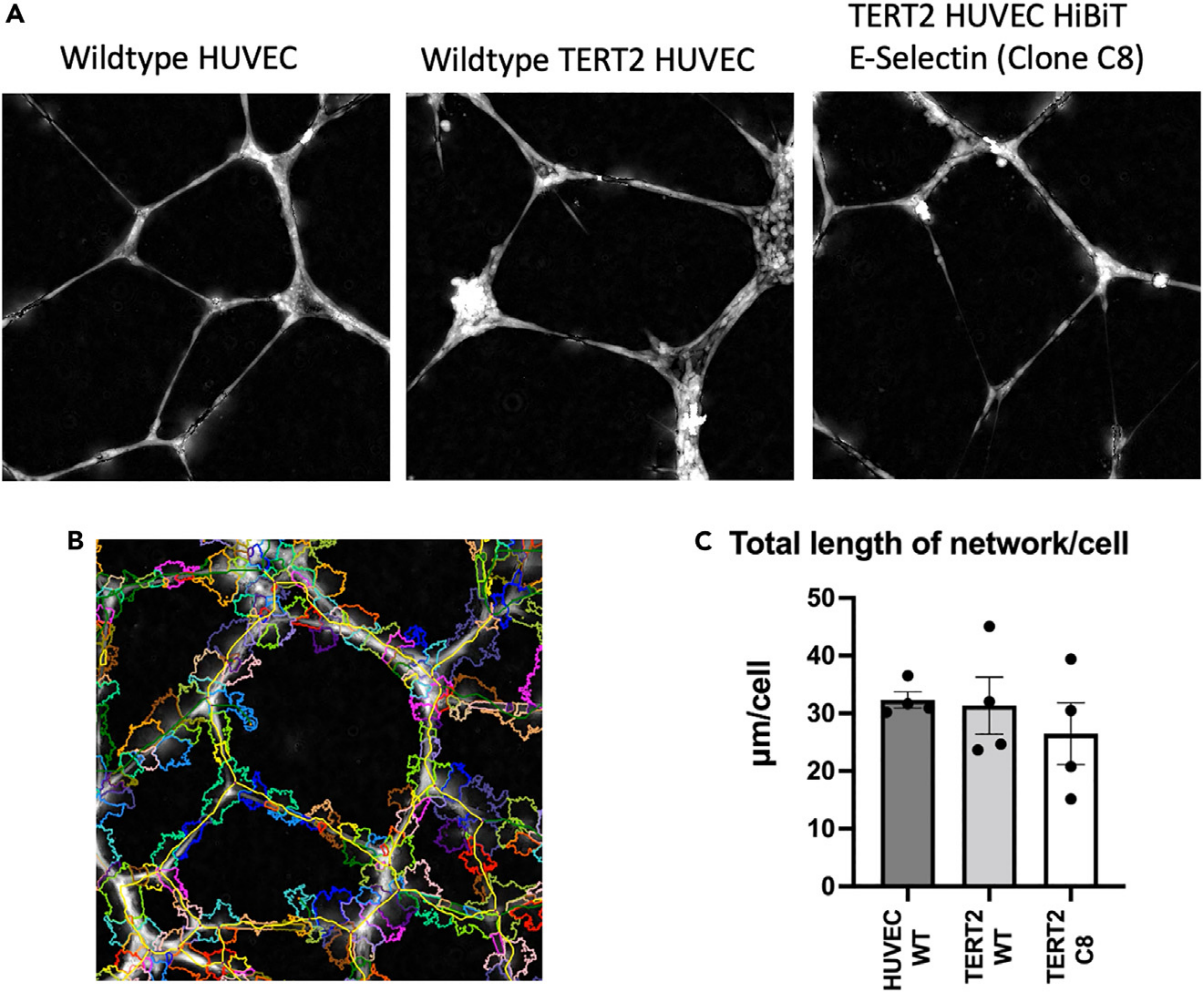
Label-free angiogenesis assays to confirm the phenotype of CRISPR-Cas9 gene-edited TERT2-HUVECs measured using the PhaseFocus Livecyte Wild-type HUVECs, wild-type TERT2-HUVECs, and CRISPR/Cas9 gene-edited TERT2-HUVECs expressing HiBiT E-selectin (clone C8) were seeded at 20,000 cells/well onto black, flat bottomed plates pre-coated with 60 μL/well Geltrex. Cells were immediately placed within the humidified LivecyteCell Imaging chamber (Phasefocus) maintained at 37°C 5% CO_2_ in air and left to equilibrate for 20 min. (A) A single 1 mm^2^ region per well was imaged with a 10× objective every hour for 10 h. Integrated angiogenesis network analysis was performed using PhaseFocus Cell Analysis Toolbox version 3.8.1 on each well over the total length of the assay. Images shown are for the 10 h time point. (B) Example of the analysis with individual cells indicated by multicolored shapes and tube networks shown in yellow. (C) The total length of the network (μm) was calculated on a per cell basis at the 10 h time point. Data are expressed as the mean ± S.E.M of 4 independent experiments (4–5 wells analyzed per cell line, per experiment).

Western blot analysis of E-selectin protein generation in response to increasing concentrations of TNFα showed similar responses in wild-type and TERT2-HUVEC cell lines (Figure S4A). Furthermore, similar responses were seen in Western blots of HiBiT-E-selectin TERT2-HUVECs probed with a HiBiT monoclonal antibody over four independent experiments (Figures S5). To ensure that the phenotype and physiological responses of wild-type HUVECs and genome-edited TERT2-HUVECs were similar, we evaluated their ability to support angiogenesis and form new blood vessels.^22^^,^^23^^,^^24^ This was demonstrated by their ability to form endothelial microtubes (microvessels) when grown in a three-dimensional (3D) matrix.^23^^,^^24^^,^^25^ Wild-type HUVECs, TERT2-HUVECS, and HiBiT-E-selectin TERT2-HUVECs were able to elicit tube formation when grown in Geltrex (Figure 5).

### Effect of VEGF_165_a and histamine on basal and TNFα-stimulated E-selectin expression

**Figure 6:**
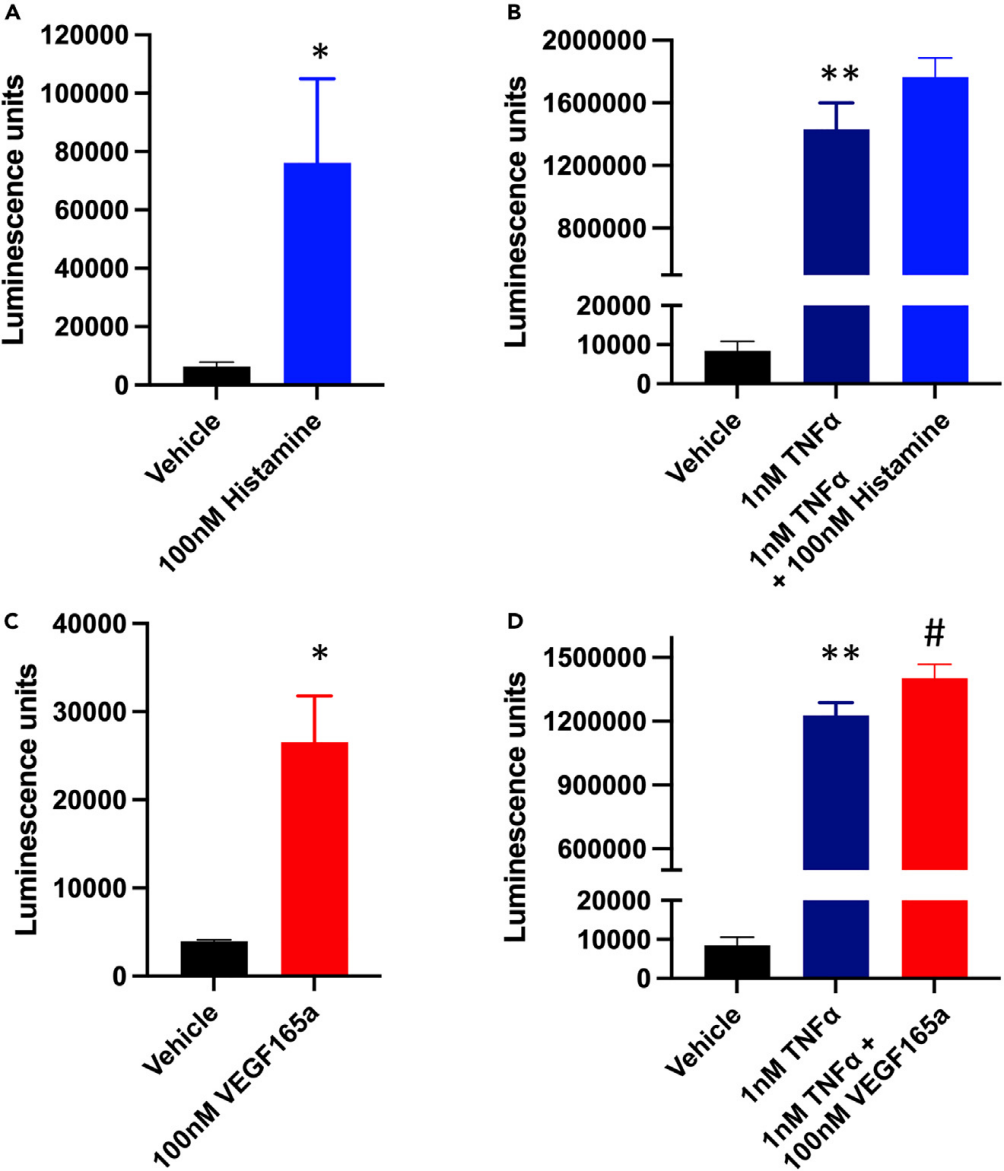
Effect of histamine and VEGF_165_a on HiBiT E-selectin expression in gene-edited TERT2-HUVEC clone C8 (A and B) Increase in luminescence under basal conditions and in response to 8 h stimulation with (A) 100 nM histamine or (B) a combination of 100 nM histamine and 1 nM TNFα in HiBiT genome-edited clone C8 TERT2-HUVECs. (C and D) Increase in luminescence under basal conditions and in response to 8 h stimulation with 100 nM VEGF_165_a (C) or (D) a combination of 100 nM VEGF_165_a and 1 nM TNFα in HiBiT genome-edited clone C8 TERT2-HUVECs. Values represent mean ± S.E.M of the five independent experiments, each performed in triplicate. ∗p < 0.05 compared to vehicle control (paired t test). ∗∗p < 0.01 compared to vehicle control or #p < 0.05 compared to TNFα alone (One-way ANOVA).

VEGF and histamine have previously been reported to either induce E-selectin expression (VEGF_165_a) or enhance TNFα-induced expression (Histamine) in vascular endothelial cells.^26^^,^^27^ In the present study, both VEGF_165_a (100 nM) and histamine (100 nM) were able to produce a significant increase in basal HiBiT-E-selectin expression (Figures 6A and 6C). Furthermore, a significant increase in the response to 1 nM TNFα was obtained in the presence of 100 nM VEGF but not histamine (100 nM) (Figures 6B and 6D). Similar effects were also seen in non-edited HUVECs using the alkaline phosphatase E-selectin antibody assay in fixed cells (Figure S6).

### Real-time kinetic analysis of TNFα-induced E-selectin expression in living cells

**Figure 7:**
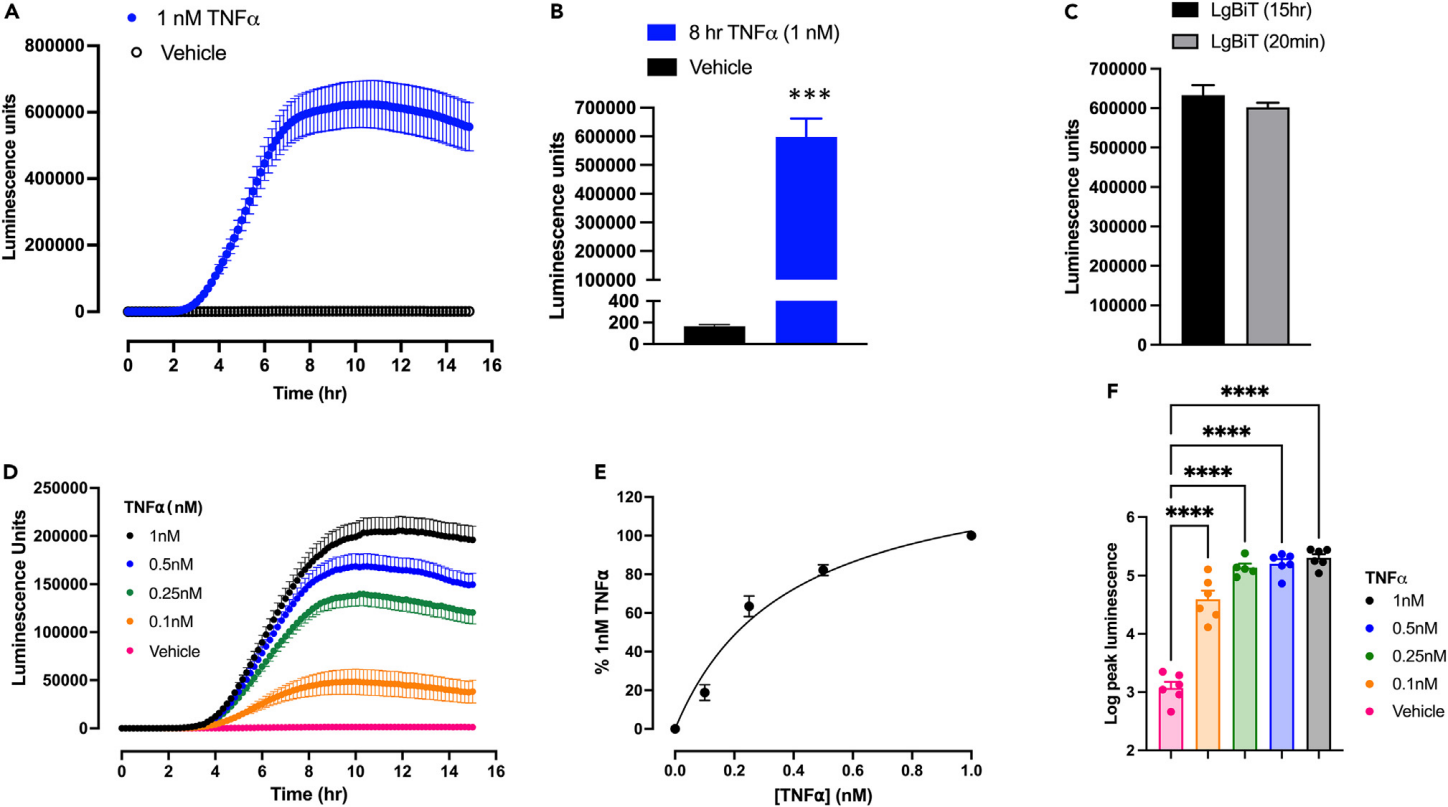
Kinetic profile of TNFα-stimulated luminescence in clonal HiBiT-edited E-selectin TERT2-HUVECs To monitor the real-time kinetics of TNFα-induced expression of E-selectin, experiments were undertaken with the homozygous TERT2-HUVEC HiBiT E-selectin clone C8. As these kinetic experiments were performed over 15 h, the long acting caged nanoluciferase substrate endurazine was used. (A) Time course of HiBiT E-selectin expression over 15 h in the continued presence of 50 nM LgBiT and endurazine. Values represent mean ± S.E.M from five independent experiments. (B) Incubation with 1 nM TNFα in the continued presence of 50 nM LgBiT and endurazine yielded a significant increase in luminescence over 8 h (∗∗∗p < 0.001; paired t test of 5 independent experiments). (C) To investigate potential depletion of LgBiT through the experiment, experiments were either performed in the continuous presence of 50 nM LgBiT incubation or following a 20 min addition of LgBiT (50 nM) at the end of the 15 h incubation with 1 nM TNFα. There was no significant difference between the luminescence values obtained. (D) Time course of HiBiT E-selectin expression in response to increasing concentrations of TNFα. Values represent mean ± S.E.M from six (or five, 0.25 nM) independent experiments. (E) Peak responses from (D) expressed as a percentage of the response to 1 nM TNFα. (F) Log peak responses to increasing concentrations of TNFα. Data taken from (E). ∗∗∗∗p < 0.0001 (one-way ANOVA with Dunnett multiple comparison test). Symbols show the individual means in 5 or 6 independent experiments.

To monitor the real-time kinetics of TNFα-induced expression of E-selectin in living cells, experiments were undertaken with the homozygous TERT2-HUVEC HiBiT E-selectin clone C8. As these kinetic experiments were performed over 15 h, the long acting caged nanoluciferase substrate endurazine was used. This is hydrolyzed at a slow rate by cellular esterases and releases furimazine throughout the experiment.^28^^,^^29^ Incubation with 1 nM TNFα resulted in a large stimulation of HiBiT-E-selectin cell surface expression that began after 2 h and peaked at 8 h (Figure 7A). The difference in the peak cell surface expression of HiBiT E-selectin between TNFα (1 nM) and vehicle-treated cells was substantial (p < 0.001; Figure 7B). The stimulation by TNFα (1 nM) at 8 h representing a 3,727-fold increase in HiBiT E-selectin expression (n = 5; Figure 7B). Over the course of the experiment, there appeared to be no depletion of the exogenously supplied purified LgBiT since there was no significant difference in the luminescence achieved with 1 nM TNFα when LgBiT was incubated for the full 15 h or added for 20 min after completion of the incubation with TNFα (Figure 7C). In a separate series of experiments, the time course of different concentrations of TNFα (0.1–1.0 nM) was investigated over 15 h (Figures 7D–7F). These data confirmed the exquisite sensitivity of this assay to monitor real-time changes in E-selectin expression over 15 h. Furthermore, the peak responses obtained from these kinetic experiments gave an EC_50_ value (0.38 nM) for TNFα which was similar to that obtained from endpoint assays (Figure 7E). Log peak luminescence values obtained at all concentrations of TNFα were significantly different from vehicle controls (p < 0.0001; one-way ANOVA with Dunnett’s multiple comparison test; Figure 7F).

## Discussion

E-selectin is a Ca^2+^-dependent C-type lectin present on the surface of endothelial cells that can interact with cell surface glycans to promote adhesion and rolling of hematopoietic cells such as circulating leukocytes and neutrophils.^2^^,^^3^^,^^4^ In the present study, we have used NanoBiT^18^^,^^19^ to investigate ligand-induced changes in the cell surface expression of this C-type lectin. To ensure that ligand-induced changes were monitored in physiologically relevant cells under the control of their native promoters, we used CRISPR-Cas9 genome editing to attach the 11 amino acid HiBiT tag^18^^,^^19^ to the N terminus of endogenous E-selectin expressed in both HUVECs and immortalized TERT2-HUVECs.^20^ Measurement of cell surface expression of HiBiT-tagged E-selectin was then made following addition of exogenous membrane-impermeable LgBiT to achieve self-complementation of the full-length nanoluciferase enzyme.^17^^,^^18^^,^^19^ For the studies in TERT2-HUVECs, we were able to isolate a homozygous clonal cell line that expressed HiBiT on both alleles.

Using this approach, we were able to demonstrate ligand-induced expression of HiBiT-tagged E-selectin in both gene-edited primary HUVECs and gene-edited immortalized TERT2-HUVECs in response to TNFα, IL-1α, IL1-β, LPS, VEGF_165_a, and histamine. The EC_50_ values for TNFα, IL-1α, IL1-β, and LPS were very similar between gene-edited primary HUVECs and the clonal gene-edited TERT2-HUVEC cell line (Table 1). Furthermore, the EC_50_ values obtained for TNFα in live gene-edited HUVECs and TERT2-HUVECs (0.06 ± 0.01 and 0.06 ± 0.02 nM, respectively) were very similar to the value obtained (0.016 ± 0.003 nM) for expression of native un-edited E-selectin in HUVECs using antibody labeling in fixed cells. Differences in the size of the HiBiT-E-selectin response to different inflammatory mediators in TERT2-HUVECs were quite noticeable with TNFα and LPS producing very large maximal responses (Figures 3E and 3F; Figure 4) while the maximal responses to IL-1α and IL-1β were much lower and accounted for circa 15% of the responses seen with TNFα and LPS (Figure 4). This probably reflects their very different mechanisms of action and receptors involved in stimulating intracellular signaling.^30^^,^^31^^,^^32^ VEGF_165_a and histamine have previously been reported to either induce E-selectin expression (VEGF_165_a) or enhance TNFα-induced expression (histamine) in vascular endothelial cells.^26^^,^^27^ In the present study, both VEGF_165_a (100 nM) and histamine (100 nM) were able to produce a small but significant increase in basal HiBiT-E-selectin expression in immortalized TERT2-HUVECs. A small increase in the response to 1 nM TNFα was also observed with VEGF_165_a but not histamine. Similar results were obtained with VEGF_165_a and histamine in fixed un-edited HUVECs using immunohistochemistry (Figure S6), although only the effect of histamine on the TNFα response was significant in the latter case.

Time course analysis of the response to TNFα in fixed HUVECs confirmed that the response required more than 1 h stimulation and that the peak was achieved following 6–8 h of stimulation with 1 nM TNFα. However, the gene-edited cells provided an opportunity to dynamically monitor expression of HiBiT-tagged E-selectin in real time in living cells. To facilitate this, we used a stabilized derivative of the nanoluciferase substrate furimazine (endurazine) that slowly releases furimazine following the action of extracellular esterases.^28^ This allowed us to monitor the expression of HiBiT-tagged E-selectin on the surface of living gene-edited TERT2-HUVECs in real time. LgBiT and endurazine were present for the full 15 h incubation and showed that E-selectin expression in response to 1 nM TNFα was increased following a 2 h lag period and reached a well-maintained plateau between 6 and 8 h. There was no evidence that LgBiT was depleted during the 15 h total incubation since the luminescence detected at the end of 15 h in the continued presence of 50 nM LgBiT was very similar to that achieved if LgBiT was added for 20 min at the end of the 15 h incubation period. Furthermore, the evaluation of low concentrations of TNFα (0.1 nM) in the kinetic assay format yielded exquisitely sensitive responses to very low concentrations of TNFα that showed very similar kinetic profiles to those obtained with higher concentrations.

In summary, the present study has shown that the use of genome editing to apply an 11 amino acid tag (HiBiT) to the N terminus of endogenous E-selectin in physiologically relevant endothelial cells, under the control of its native promoter, provides a powerful way to monitor ligand-induced changes in this important inflammatory cell surface protein. We have demonstrated the power of this approach by showing that E-selectin can be induced in real time in living cells by a range of different inflammatory mediators. This gene-editing approach therefore represents a powerful tool to aid mechanistic analysis of future drug discovery efforts aimed at inflammatory diseases. Furthermore, the kinetics studies in live cells made possible by the genome-edited HiBiT-E-selectin TERT2-HUVECs provide an exquisitely sensitive method to monitor changes in E-selectin in response to inflammatory mediators in living cells and in real time.

### Limitations of the study

The use here of NanoBiT in conjunction with CRISPR-Cas9 gene editing allowed the real-time quantification of cell surface E-selectin expression on HUVECs. However, a limitation of using this technique in wild-type HUVECs (which have a limited window of passages before phenotypic changes are observed) was the inability to clone these cells to produce a homogeneous population of cells positive for the HiBiT insertion. This meant that each experimental replicate required its own electroporation to deliver the CRISPR-Cas9 reagents to the HUVEC. This factor, combined with the inherently lower editing efficiency typically seen for “knock-in” edits, resulted in decreased luminescence outputs more influenced by any variability in cell seeding. To address this issue, we generated a CRISPR-Cas9 TERT2-HUVEC clone HiBiT E-selectin (clone C8). This required single-cell seeding of a mixed population of immortalized TERT2-HUVECs and screening of clones for expression and homozygosity of HiBiT insertion, which can be a lengthy process. The clone we generated was homozygous for the HiBiT edit, resulting in considerably greater experimental windows with more consistent luminescence output per experiment. This allowed detailed live cell kinetics of E-selectin to be monitored in real time and provides a powerful tool for future drug discovery efforts at this important inflammatory protein. Future drug-screening hits can then be confirmed in wild-type HUVECs and related endothelials cells using CRISPR-Cas9 editing. Phenotypic validation (e.g. CD31 immunolabeling or endothelial microtubes (microvessels) formation when grown in a 3D matrix) should, however, always be performed for all edited cell populations (both mixed and clonal) and/or following dilution cloning. The approach used here was performed under a materials transfer agreement for non-commercial use from ATCC. Any future drug-screening application will, however, require an additional commercial use license from ATCC.

## STAR★Methods

### Key resources table

**Table:** 

REAGENT or RESOURCE	SOURCE	IDENTIFIER
**Antibodies**		

Anti-E-Selectin (CD62E) antibody, Mouse monoclonal	Sigma Aldrich (UK)	Cat# S9555; RRID: AB_1078476
Chicken anti-Mouse IgG (H+L) Cross-Adsorbed Secondary Antibody, Alexa Fluor™ 488	ThermoFisher Scientific (USA)	Cat# A-21200; RRID: AB_2535786
Anti-Mouse IgG (whole molecule)−Alkaline Phosphatase antibody produced in goat	Sigma Aldrich (UK)	Cat# A9316; RRID: AB_258446
Anti-HiBiT antibody, mouse monoclonal	Promega (USA)	Cat# N7200; RRID: AB_2924793
Anti-α-tubulin antibody, rabbit monoclonal	Cell Signalling Technologies (The Netherlands)	Cat# 2144; RRID: AB_2210548
IRDye 800CW goat anti-mouse IgG secondary antibody (H+L)	Li-Cor Biosciences (UK)	Cat# 925-32210; RRID: AB_2687825
IRDye 680RD goat anti-rabbit IgG secondary antibody (H+L)	Li-Cor Biosciences (UK)	Cat# 925-68071; RRID: AB_2721181

**Chemicals, peptides, recombinant proteins**		

FuGENE HD Transfection Reagent	Promega (USA)	Cat# E2311
Opti-MEM reduced serum medium	ThermoFisher Scientific (USA)	Cat# 11058021
Formalin solution neutral buffered, 10%	Sigma Aldrich (UK)	Cat# HT501128
Chicken serum	Sigma Aldrich (UK)	Cat# C5405
Goat serum	Abcam (UK)	Cat# ab7481
Bovine serum albumin (BSA)	Sigma Aldrich (UK)	Cat# 03117332001
Glycine	Sigma Aldrich (UK)	Cat# G8898
bisBenzimide H33342 trihydrochloride	Sigma Aldrich (UK)	Cat# B2261
Human Large Vessel Endothelial Cell Basal Medium (Medium 200)	ThermoFisher Scientific (Gibco) (USA)	Cat# M200500
Large Vessel Endothelial Supplement (LVES)	ThermoFisher Scientific (Gibco) (USA)	Cat# A1460801
Dulbecco’s Modified Eagle’s Medium (DMEM)	Sigma Aldrich (UK)	Cat# D6429
Fetal Calf Serum (FCS)	Sigma Aldrich (UK)	Cat# F2242
Trypsin-EDTA Solution x10	Sigma Aldrich (UK)	Cat# T4174
Dulbecco's Phosphate Buffered Saline	Sigma Aldrich (UK)	Cat# D8537
Poly-D-Lysine hydrobromide	Sigma Aldrich (UK)	Cat# P6407
Gelatin solution	Sigma Aldrich (UK)	Cat# G1393
LB Broth (Lennox)	Sigma Aldrich (UK)	Cat# L3022
Tumor Necrosis Factor-α human	Sigma Aldrich (UK)	Cat# H8916
Recombinant Human VEGF 165 Protein (with carrier)	R&D systems (USA)	Cat# 293-VE-050
Histamine dihydrochloride	Sigma Aldrich (UK)	Cat# H7250
IL-1 alpha human	Sigma Aldrich (UK)	Cat# SRP6295
IL-1 beta human	Sigma Aldrich (UK)	Cat# SRP3083
Lipopolysaccharides from Escherichia coli O55:B5	Sigma Aldrich (UK)	Cat# L2880
RIPA buffer	Sigma Aldrich	Cat# R0278
Roche cOmplete, mini protease inhibitor cocktail	Sigma Aldrich	Cat# 11836153001
Pierce™ BCA Protein Assay Kit	ThermoFisher (UK)	Cat# 23227
Invitrogen NuPAGE™ LDS Sample Buffer	ThermoFisher (UK)	Cat# NP0007
Invitrogen NuPAGE™ Sample Reducing Agent	ThermoFisher (UK)	Cat# NP0004
Invitrogen NuPAGE™ MES SDS Running Buffer	ThermoFisher (UK)	Cat# NP0002
Invitrogen Nitrocellulose/Filter Paper Sandwich, 0.45 μm, 8.3 x 7.3 cm	ThermoFisher (UK)	Cat# LC2001
Invitrogen NuPAGE™ Transfer Buffer	ThermoFisher (UK)	Cat# NP00061
Intercept (TBS) blocking buffer	Li-Cor Biosiences (UK)	Cat# 927-60001
Li-CoR Total Protein Stain	Li-Cor Biosiences (UK)	Cat# 926-11010
GeltrexTM LDEV-free reduced growth factor basement membrane matrix	ThermoFisher Scientific (USA)	Cat# A1413202
Purified Cas 9	IDT (USA)	Cat# 1081059
Electroporation enhancer	IDT (USA)	Cat# 1075916
Ingenio® Electroporation Solution	Mirus Bio (USA)	Cat# MIR 50114

**Critical commercial assays**		

Nano-Glo luciferase assay (Furimazine)	Promega (USA)	Cat# N1130
Nano-Glo HiBiT Lytic Detection System	Promega (USA)	Cat# N3040

**Experimental models: Cell lines**		

Human umbilical vein endothelial cells (HUVECs) (newborn male single donor)	ThermoFisher Scientific (USA)	Cat# C0035C Lot#: 1606186
Human telomerase reverse transcriptase 2 (hTERT2) HUVECs (female)	ATCC (Virginia, USA)	Cat# CRL-4053 Lot# 70023166

**Oligonucleotides**		

E-selectin guide DNA	IDT (USA)	Custom
TRACR RNA	IDT (USA)	Cat# 1073191
HiBiT E-selectin Repair DNA	IDT (USA)	Custom

**Recombinant DNA**		

E-selectin vector	Vector Builder (USA)	Custom

**Software and algorithms**		

GraphPad Prism 7.02	GraphPad Software	www.graphpad.com
Zen 2012	Zeiss	www.zeiss.com
ImageJ Fiji 1.53	National Institute of Health	www.fiji.sc

**Other**		

μ-Slide 8 well plate - ibidiTreat: #1.5 polymer coverslip, tissue culture treated, sterilized	Thistle Scientific (Ibidi)	Cat# 80826
Greiner 96 well plate (White)	Greiner Bio-One	Cat# 655089
Greiner 96 well plate (Black)	Greiner Bio-One	Cat# 655090
Corning® 96-well Clear Flat Bottom Polystyrene TC-treated Microplate	Corning (USA)	Cat# 3596
4-chamber 35mm dish	Cellvis (USA)	Cat# D35C4-20-1.5-N
Fisherbrand™ Electroporation Cuvettes Plus™	Fisher scientific	Cat# FB102
Nucleofector® 2b Device	Lonza	Cat# AAB-1001
E.Z.N.A.® Tissue DNA Kit	Omega Bio-tek (USA)	Cat# D3396-01
Wizard SV Gel and PCR Clean-Up System	Promega (USA)	Cat# A9281
BamHI enzyme	Promega (USA)	Cat# R6021
XhoI enzyme	Promega (USA)	Cat# R6161
DH5 alpha Chemically Competent cells	invitrogen	Cat# 18265-017
T4 DNA ligase	New England Biolabs	Cat# MO202S

### Resource availability

#### Lead contact

Further information and requests for resources and reagents should be directed to and will be fulfilled by the Lead Contact, Stephen J Hill (stephen.hill@nottingham.ac.uk).

#### Materials availability

Materials developed from this study are available from the lead author on reasonable request.

### Experimental model and study participant details

Male human umbilical vein endothelial cells (HUVECs) and female human TERT2 immortalised HUVECs were used in this study.

### Method details

#### Cell culture

All cell types were grown at 37°C/5% CO_2_ and passaged when they reached 70% confluency using phosphate buffered saline (PBS; D8537; Lonza, Germany) trypsin (0.25% w/v or 1% w/v in versine; T4174; Sigma Aldrich). Human umbilical vein endothelial cells (wildtype HUVECs; newborn male, single donor) were obtained from ThermoFisher Scientific (C0035C. Lot number: 1606186). Human TERT2 immortalised HUVECs (hTERT2-HUVECs; female, neonate) were obtained from ATCC (CRL-4053; LOT# 70023166). HUVECs and TERT2-HUVECs were cultured in Medium 200 (M200500) supplemented with 2.2% large vessel endothelial supplement (LVES; A14608-01) both purchased from ThermoFisher Scientific, UK. TERT2-HUVECs were grown on 0.1% gelatin coated tissue culture flasks (G1393; Sigma Aldrich). Wildtype HUVECs were used up to passage 6, whilst TERT2 immortalised HUVECs were used up to passage 19.

#### Cell line authentication

TERT2 immortalised HUVECs and HiBiT edited TERT2 immortalised HUVECs (C8) were grown to confluence and detached with trypsin. Cells were pelleted and washed twice with PBS. Short tandem repeat (STR) cell line authentication^21^ of wild-type and genome-edited hTERT2-HUVECs was performed by Eurofins using 16 DNA markers with the Applied Biosystems™AmpFLSTR™ Identifiler™ Plus PCR amplification kit system. Analysis of STR profiles was performed using www.cellosaurus.org.^33^ and the Cellosaurus STR similarity search tool (CLASTR).^34^ A cut off of 80% was used as a threshold for declaring a genetic match with an original online cell line profile to account for genetic drift.^21^

#### Western blot analysis

Cells were plated in 12 well plates and grown to confluence. On the day of experiment, growth media was replaced, and cells treated with TNFα (0, 0.05, 0.5, 1 nM), for 6 hours. At the end of experimental treatments, cells were washed twice with ice cold PBS, and lysed with RIPA buffer (R0278, Sigma-Aldrich), supplemented with cOmplete™, Mini Protease Inhibitor Cocktail (Roche), and collected in microcentrifuge tubes. Samples were incubated at 4°C for 30 minutes with constant agitation, before centrifugation at 13,000 g for 15 minutes at 4°C, supernatants were retained for analysis. Protein concentration was determined utilising the Pierce™ BCA Protein Assay Kit (Thermo Scientific) according to manufacturer’s instructions. Protein concentration was normalised between samples before addition of NuPAGE™ LDS Sample Buffer (NP0007, Invitrogen). In samples to be probed with anti HiBiT antibody, samples were supplemented with NuPAGE™ Sample Reducing Agent (NP0004, Invitrogen) and incubated at 70°C for 10 minutes.

9 μg of protein was separated on 4-12% Bis-Tris gels (NP0323, Invitrogen) by electrophoreisis at 200 V for 35 minutes in NuPAGE™ MES SDS Running Buffer (NP0002, Invitrogen). Proteins were transferred to 0.45 μM nitrocellulose membranes (LC2001, Invitrogen) by wet transfer in NuPAGE™ Transfer Buffer (NP00061, Invitrogen) at 30 V for 70 minutes. Membranes were blocked by incubation in Intercept® (TBS) Blocking Buffer (927-60001, Li-Cor) for 1 hour at room temperature. Membranes were incubated in primary antibodies diluted in blocking buffer supplemented with 0.2% v/v Tween20 overnight (∼ 16 hours ) at 4°C with constant agitation. Primary antibodies used were mouse monoclonal anti-HiBiT (N7200, Promega; 1:1000 dilution), mouse monoclonal anti-E-selectin (CD62E; 1:1000 dilution) and rabbit anti-α-tubulin (2114; Cell Signalling; 1:1000 dilution). Protein standards were run in parallel (Precision Plus Protein All Blue prestained protein standards (BioRad).

Following incubation with primary antibody, membranes were washed 3 x 5 minutes in Tris-Buffered Saline (TBS) supplemented with 0.1% Tween 20 (TBS-T). Membranes were then incubated with secondary antibodies diluted in blocking buffer supplemented with 0.2% Tween 20, for 1 hour at room temperature. The secondary antibodies used (1:10,000 dilution) were IRDye® 680RD Goat anti-Rabbit IgG (H + L) or IRDye® 800CW Goat anti-Mouse IgG (H + L). Following secondary antibody incubation, membranes were washed 2 x 5 minutes in TBS-T and a further 5 minutes in TBS. Blot images were obtained using a Li-Cor Odyssey scanner. To confirm equal protein transfer, the E-selectin blot was also stained with Li-CoR 700 Total Protein stain for 5 min, rinsed in doble distilled water, and imaged using a Li-CoR Odyssey scanner.

#### Confocal imaging

Wildtype HUVECs were seeded onto an ‘ibidiTreat’ coated μ-Slide 8 well plate (Ibidi, IB-80821; Thistle Scientific, UK) at 10,000 cells/well and incubated for 24h (37°C/5% CO_2_). The following day, wells were treated with either TNFα (1nM), VEGF_165_a (100nM) or Histamine (100nM) for 8h, 6h, 4h, 1h and 30 min, before cells were fixed with 3% paraformaldehyde (PFA; HT501128; Sigma Aldrich) in PBS (20min, RT). Wells were washed between each step 3 times (5min, RT) with PBS. Cells were blocked with 3% bovine serum albumin (BSA) supplemented with 1% glycine (G8898; Sigma Aldrich) in PBS for 30min at room temperature, followed by 10% chicken serum in PBS (30min, room temperature). Cells were then labelled using Anti-E-selectin (CD62E) mouse monoclonal antibody overnight at 4°C (1:2000 dilution). The next day, cells were extensively washed using PBS, then labelled using a chicken anti-mouse secondary antibody conjugated to AlexaFluor 488 (1:500 dilution in 10% chicken serum/PBS; 1h at room temperature) and nuclei labelled using bisBenzimide H 33342 trihydrochloride (H33342; 1:1000 dilution in PBS, 20min at room temperature). Wells were left in PBS before imaging using a Zeiss LSM880 confocal microscope fitted with a 40x/1.20W Corr M27 water-immersion objective (Zeiss, Germany). An Argon 488 / Diode 405-30 laser was used, at 2% laser power with a pinhole diameter of 1 airy unit (AU). Image acquisition was performed using the proprietary Zeiss software Zen, at 512 x 512 frame size, with 8 averages, and gains of 850 (for 488nm excitation) and 600 (for 405nm excitation). Images were edited with the addition of scale bars and exported as 8-bit TIFs using Zen 2010 software (Zeiss, Germany).

#### Time course and concentration response quantification using alkaline phosphatase

Wildtype HUVECs were seeded onto clear 96-well Clear Flat Bottom Polystyrene tissue culture-treated Microplates (3596; Corning, USA) at 30,000 cells/well and incubated for 24h (37°C, 5% CO_2_). The following day, cells were incubated with 100μl Medium 200, with increasing concentrations of TNFα (0-1nM) or fixed concentrations of VEGF_165_a (100nM) or histamine (100nM) (6h 37°C 5% CO_2_). For time course assays cells were incubated for 8h, 6h, 4h, 1h and 30min with TNFα (1nM (37°C 5% CO2). Cells were fixed and primary antibody labelled as described above. The following day, cells were secondary antibody labelled with Anti-Mouse IgG (whole molecule) Alkaline Phosphatase antibody produced in goat (1h, room temperature). After 5 washes with PBS, wells were incubated with 100μl 3.7% (w/v) p-Nitrophenyl Phosphate substrate (pNPP) (Thermofisher Scientific USA, 34045), diluted in 1 litre diethanolamine (DEA) buffer (16.36g NaCl, 102mg MgCl_2_ 6 H_2_O, 100ml DEA, in 1L ddH_2_O, pH 9.85) for 20min (at room temperature). The plate was read immediately using a Dynex MRX Revelation Absorbance Micro Plate Reader at absorbance 405nm.

#### CRISPR/Cas9 genome editing

HUVEC genomic DNA was isolated and checked for single nucleotide polymorphisms (SNPs) after DNA extraction using E.Z.N.A Tissue DNA Kit (D3396-01; Omega Bio-tek, USA) polymerase chain reaction (PCR) analysis and Sanger sequencing (DEEPSEQ, University of Nottingham). Briefly, a 379 bp region of the N-terminal region of E-selectin was sequenced following amplification by PCR using the forward primer 5’-GAGCCCAGTTCTTGGCTTCT-3’ and the reverse primer 5’-TCTGGGCCATGTCACAAACA-3’.

CRISPR RNA (crRNA) was designed to be homologous to the N-terminal region of interest on the N-terminus of E-selectin (CGTGGAGGTGTTGTAAGACC) immediately after its signal sequence (TGCTTCTCATTAAAGAGAGTGGAGCC). The sequences were designed using the CRISPOR program design tool (http://crispor.tefor.net), by inputting this N-terminal section of E-selectin genomic sequence (found on University of California Santa Cruz (UCSC) using the latest upload; Dec.2013 (GRCh38/hg38 human assembly) into the software.^35^ Potential guide sequences for protospacer adjacent motif (PAM) regions were listed and the highest predicted efficiency sequence and location were selected. Cas9 protein requires a PAM site (specifically NGG for Cas9, where ‘N’ is any nucleotide base) to serve as a binding signal for Cas9. The selected guide sequence had an MIT specificity score of 88, indicating a low likelihood of off-target effects.^36^ The complete RNA guide sequence used was 5’-**CGUGGAGGUGUUGUAAGACC**GUUUUAGAGCUAUGCU-3’ (with the target sequence in bold).

The repair template was designed to contain the desired HiBiT sequence (GTGAGCGGCTGGCGGCTGTTCAAGAAGATTAGC), with a Glycine-Serine-Serine-Glycine (GSSG) linker (GGGAGTTCTGGC), containing left and right homology arms (71 base pairs in length) on either side to aid the insertion of the HiBiT-GSSG sequence.

The sequence used was

5’CAACAGTACCAAACTCTACCATTTCTTTTCTTTTTCTCCCACTAGTGCTTCTCATTAAAGAGAGTGGAG**CC**GTGAGCGGCTGGCGGCTGTTCAAGAAGATTAGCgggagttctggc**T**GGTCTTACAACACCTCCACGGAAGCTATGACTTATGATGAGGCCAGTGCTTATTGTCAGCAAAGGTACAC-3’. Within the repair template, the PAM site (CCT in bold) was disrupted by the insertion of the HiBiT (underlined) and the GSSG linker (lower case) sequences to prevent re-cutting by residual Cas9. The repair template was synthesized as a single stranded oligo DNA nucleotide (ssODN). All CRISPR/Cas9 reagents (including the tracrRNA) were purchased from Integrated DNA Technologies, Inc. (IDT; Iowa, USA).

#### Generation of HiBiT E-selectin mixed population of wildtype and TERT2 HUVECs

48h before, HUVECs or TERT2 HUVECs were passaged to attain 80% density at the time of electroporation. On the day of electroporation, HUVECs or TERT2-HUVECs were treated with trypsin (1% w/v in versine) and pelleted. Cells (500,000) were then re-pelleted before resuspending in 100μl warm Ingenio Electroporation Solution. The ribonucleoprotein (RNP) complex was formed by annealing equal parts E-selectin guide RNA and tracrRNA for 5 min at 95°C, before addition of purified Cas9 protein and sterile PBS (20 min at room temperature) to achieve a 2:1 ratio of guide RNA:Cas9 protein (1500nM guideRNA:750nM Cas9 final concentration in electroporation cuvette). The RNP mix was then incubated with HiBiT E-selectin repair template and Electroporation Enhancer, before mixing with resuspended cells gently but thoroughly. Using the Ingenio plastic cell dropper, the mixture was gently transferred into the electroporation cuvette avoiding bubbles. Cells were then electroporated using Nucleofector 2b Device electroporator (HUVEC - human A-034 setting). CRISPR/Cas9 edited HUVEC cells were then resuspended in an appropriate volume of warm Medium 200 before they were seeded at 20,000 cells per well in 0.1% gelatin coated white sided, flat bottomed Greiner 96 well plate (Greiner Bio-one; 655098), containing 50μl pre-warmed Medium 200. Medium 200 was replaced 24h after electroporation. Gene-edited TERT2 HUVECs were resuspended in warmed Medium 200 supplemented with 2.2% LVES and grown in a 25cm^2^ tissue culture flask (T25) which had been pre-coated with 0.1% gelatin (20min, 37°C). Media was replaced every 2 days to aid growth of HiBiT E-selectin mixed population (mx) TERT2 HUVECs. When cells reached 70-80% confluency, cells were transferred into a T75 flask.

#### Generation of a HiBiT E-selectin TERT2 HUVEC clonal cell line

To generate a clonal HiBiT E-selectin TERT2 HUVEC cell line, HiBiT E-selectin (mixed population) TERT2 HUVECs were single cell seeded into a 0.1% gelatin coated 96 well plate. This was achieved by diluting the cell population to give a density of 50 cells per ml. Cells were seeded at 50μl cell suspension per well, supplemented with 150 μl Medium 200 and left to grow over the course of 3-4 weeks. Each well was visually checked for single colonies. Media was replaced to aid the growth of cells and minimise the effect of any well evaporation. When the single seeded TERT2 HUVECs had grown to >60% confluency in a 96 well plate, cells were lifted off each well by addition of 50μl 4X Trypsin (< 5min 37°C). 100μl Medium 200 was then added per well and half of the volume was reseeded in a duplicate 0.1% gelatin coated white-sided, flat bottomed Greiner 96 well plate. The rest of the cells were left in the plate to adhere and regrow, for further use. Media was replaced the following day after cells had adhered to both 96 well plates to promote cell growth. When the reseeded cells for screening grew to >70% confluency, every well was incubated with 1nM TNFα diluted in Medium 200 supplemented with 2.2% LVES (6h, 37°C 5% CO_2_). Purified LgBiT protein (N3030, Promega USA) was then added (50nM final concentration in well, 37°C, 20min) and Furimazine substrate (N1110, Promega USA) was added (1:400 final dilution in well, 5min) before an opaque plate back was added (Perkin Elmer) and luminescence measured using a PHERAstar FS plate reader (BMG LabTech) using filters to measure NanoLuc emissions at 450 nm (30nm bandpass). ‘Hit’ wells were defined as wells producing luminescence units of >100,000. ‘Hit’ wells were then identified in the corresponding original 96 well plate and expanded for further study.

#### Verification of homozygous edit

To test whether one allele (heterozygous) or both alleles (homozygous) of the E-selectin gene had been edited with the HiBiT tag, PCR and gel electrophoresis techniques were used. A 370 bp N-terminal region of E-selectin was amplified by PCR using the primers described above. If the HiBiT edit had been successful, a 45bp addition (HiBiT + GSSG linker) in length would be visible on an agarose gel compared to WT unedited DNA. If the edit was heterozygous, two bands would be observed, indicative of a population of unedited (370bp) and edited (415bp) DNA. If the edit was homozygous, only one band would be observed on the agarose gel (415bp). TERT2 HUVEC clone C8 demonstrated a homozygous addition of HiBiT + GSSG linker and was used for further studies. The HiBiT E-selectin clone DNA and wildtype TERT2 HUVEC DNA were isolated using the E.Z.N.A Tissue DNA Kit, and the N-terminal region of E-selectin was then amplified using PCR as described above. Amplified DNA was run on a 3% agarose gel at 90 V for 1 hour using a 100bp DNA ladder for reference (N3231S, New England BioLabs UK).

#### Agonist-stimulated HiBiT E-selectin expression in gene-edited HUVECs

Concentration responses for TNFα, lipopolysaccharide (LPS), interleukin-1 alpha (IL-1α) and interleukin-1 beta (IL-1β) were conducted in genome edited WT HUVECs and TERT2 HUVECs. All genome edited HUVECs/TERT2 HUVECs were seeded onto 0.1% gelatin coated white-sided 96 well plates. For edited WT HUVECs, cells were seeded at 20,000 cells per well. For genome edited TERT2 HUVECs (mixed population and clonal cell lines), cells were seeded at 30,000 cells per well. All experimentation was undertaken when cells reached 80% confluency in each well. On the day of experimentation, TNFα (dose range (nM): 0.05, 0.50, 1.0), LPS (dose range (nM): 5.0, 30, 50), IL-1α and IL-1 β (dose range (nM): 50, 200, 500) were diluted to their appropriate concentrations in Medium 200 supplemented with 2.2% LVES. HiBiT-GSSG genome edited WT HUVECs, TERT2 HUVECs (mixed population) and clonal TERT2 HUVECs were incubated with the appropriate doses of TNFα, LPS, IL-1α and IL-1β for 6 hours (37°C, 5% CO_2_). NanoBiT complementation was then conducted by addition of LgBiT (50nM) (20 min, 37°C) and furimazine substrate (1:400), and the plates were read with an opaque plate back added using a BMG PHERAstar FS plate reader (450 nm, 30nm bandpass).

To evaluate the time-course of E-selectin expression in HiBiT E-selectin edited TERT2 HUVECs in response to TNFα over 15 hours, experiments were conducted in the presence of a caged nanoluciferase substrate (Endurazine™; N2570, Promega USA) in Opti-MEM medium. Clonal HiBiT-TERT2-HUVECs (clone C8) were seeded onto 0.1% gelatin coated white-sided 96 well plate at 30,000 cells/well and experimented on 24 h later. On the day of experimentation, all appropriate wells were replaced with Opti-MEM, supplemented with Endurazine (1:100), and LgBiT (50nM). Wells were included where there was no addition of LgBiT (LgBiT control wells) to test for LgBiT depletion. 50μl PBS was then added to all other wells to aid with humidity control across the plate. TNFα (1nM) was then added into the appropriate wells in triplicate. Kinetic experiments were also performed using the same experimental protocol but with 0.1, 0.25, 0.5 or 1nM TNFa added in quadruplicate wells. An Empore™ Sealing Tape (66881-U, Sigma Aldrich) was adhered to the top of the plate to prevent evaporation of buffer from the wells throughout the 15h kinetic screen, and a plate back was adhered to the bottom of the plate. The plate was then immediately put into the BMG PHERAstar FS plate reader, set at 37°C to read luminescence at 450nm (30nm bandpass, gain 3600) every 10 minutes for 15 hours. The next day, LgBiT (50nM) was added to the LgBiT control wells, and luminescence output was compared to vehicle wells (where there was no cytokine addition, 1:100 Endurazine and 50nM LgBiT, (incubated for 15 h 37°C)) by addition of 1 nM purified HiBiT protein (N3010, Promega USA) (5min, 37°C).

#### Bioluminescence microscopy

Genome edited HiBiT E-selectin HUVEC/TERT2 HUVEC cells were imaged for luminescence using a live cell bioluminescence imaging system; Olympus Luminoview 200 (LV200, Olympus Lifescience) fit with a PanApochromat 60x NA1.42 oil immersion objective with 0.5x tube length, resulting in 30x final magnification. Cells were plated onto a Cellvis 4-chamber 35mm dish (NC0832919, Fisher Scientific, UK), at 100,000 cells per quadrant overnight (37°C, 5% CO_2_). HiBiT E-selectin expression was induced by 1nM TNFα (6h, 37°C 5% CO_2_) and NanoBiT complementation was then induced after addition of purified LgBiT protein (50nM) in 0.1% BSA HBSS for 20min (37°C). Furimazine was then added (1:400 in 0.1% BSA HBSS) (5min, RT) and 512 x 512 images were captured using brightfield (exposure 50 ms, gain 50) and bioluminescence filters (exposure 20s, gain 200).

#### PhaseFocus Livecyte™Cell Imaging angiogenesis assay

A black, clear flat bottomed, 96-well plate (#3603, Corning®) was placed over a cold metal block, pre-chilled in a -80°C freezer, and pre-coated with 60 μl/well Geltrex™ (Geltrex™ LDEV-Free Reduced Growth Factor Basement Membrane Matrix). The plate was then left for an 1 hour at 37°C/5% CO_2_ to allow matrix polymerisation before addition of 50 μl pre-warmed Medium 200 supplemented with 2.2% LVES for a further 20 min at 37°C/5% CO_2_. Wildtype HUVECs, wildtype TERT2-HUVECs or TERT2 HUVECs CRISPR/Cas9 gene edited to express HiBiT E-Selectin (Clone C8) were seeded at 20,000 cells per well in Medium 200/2.2% LVES. The seeded plate was immediately placed within the humidified Livecyte™Cell Imaging chamber (Phasefocus™) maintained at 37°C 5% CO_2_ in air and left to equilibrate for 20 min. A single 1mm^2^ region per well was imaged with a 10x objective every hour for 12 hours. Angiogenesis network analysis was performed using PhaseFocus™ Cell Analysis Toolbox version 3.8.1.

### Quantification and statistical analysis

All quantified data are expressed as a mean ± standard error from the mean (SEM) and presented and analysed using GraphPad Prism software version 9.4.1. (GraphPad Software, LLC).

Concentration-response curves for agonist-stimulated E-selectin expression were fitted to the following equation:

$$Increase\;in\;luminescence\;intensity=\frac{E_{\max}.\left[A\right]}{\left(\left[A\right]+{EC}_{50}\right)}$$

Where E_max_ is the maximum response to the cytokine under study, [A] is the concentration of cytokine and EC_50_ is the concentration of cytokine required to produce 50% of its maximum response.

Statistical analysis was performed using paired tests or one-way ANOVA with Dunnett multiple comparison test. Statistical significance was taken as p<0.05. Statistical details of experiments can be found in the figure and table legends.

## Data Availability

• All data reported in this paper will be shared by the lead contact upon request. • This study did not generate datasets or code. • Any additional information required to reanalyze the data reported is available from the lead contact upon request.
