# Supplementary material for: Probing expression of E-selectin using CRISPR-Cas9-mediated tagging with HiBiT in human endothelial cells
Source: iScience. 2023 Jun 30;26(7):107232. doi: 10.1016/j.isci.2023.107232 (PMC10366498; doi:10.1016/j.isci.2023.107232)
Supplement: Document S1. Figures S1–S6 and Table S1 [file mmc1.pdf]

iScience, Volume *26*

Supplemental information

Probing expression of E-selectin using CRISPR-Cas9-mediated tagging with HiBiT in human endothelial cells

Lydia Ogrodzinski, Simon Platt, Joelle Goulding, Cameron Alexander, Tracy D. Farr, Jeanette Woolard, Stephen J. Hill, and Laura E. Kilpatrick

Supplementary Information

Table S1. Short tandem repeat (STR) genetic profile of wild-type and HiBiT-E-selectin gene-edited TERT2-HUVECs. Related to Figure 2.

**Short tandem repeat (STR) target loci**	**TERT2-HUVEC**	**HiBiT E-selectin gene-edited TERT2-HUVEC clone C8**
D8S1179	13,14	13,14
D21S11	28,31.2	28,31.2
D7S820	9,11	9,11
CSF1PO	12,12	12,12
D3S1358	15,16	15,16
TH01	6,9	6,9
D13S317	11,12	11,12
D16S539	9,13	9,13
D2S1338	20,25	20,25
D19S433	12,14	12,14
vWA	16,19	16,19
TPOX	8,9	8,9
D18S51	13,17	13,17
AMEL	X,X	X,X
D5S818	11,12	11,12
FGA	23,24	23,24

Results from STR genetic profiling by Eurofins using 16 DNA markers with the Applied Biosystems™AmpFLSTR™ Identifiler™ Plus PCR amplification kit system. The data were also 86.7% identical to the original TERT2-HUVEC cell deposited with ATCC (CRC-4053).

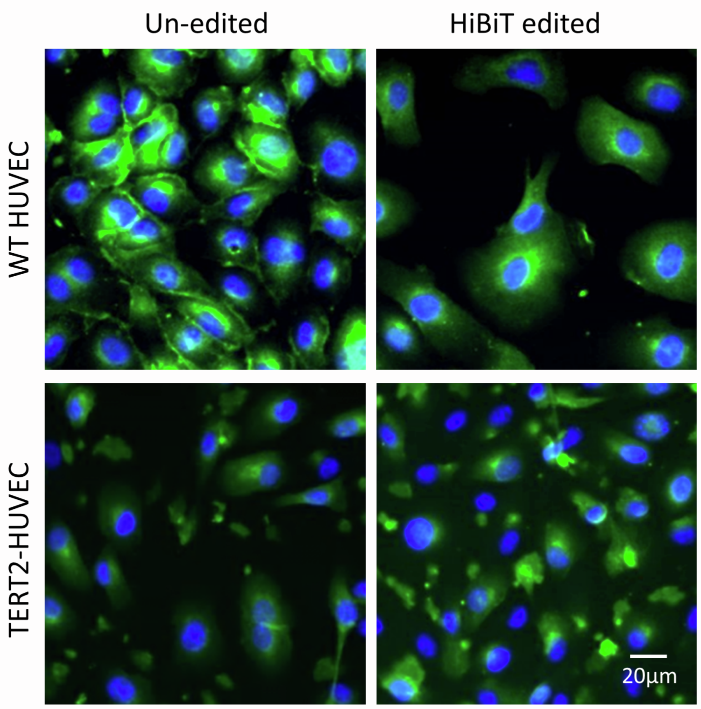

Figure S1. Effect of genome editing on endothelial cell phenotype. Related to Figures 2 and 5. HiBiT-E-selectin gene-edited WT HUVECs and TERT2 HUVECs were labelled for CD31 expression using an anti-CD31 primary antibody and Alexa Fluor-488 secondary antibody. CD31 expression was compared for HiBiT edited or unedited cells. Blue signal = H33342 labelled nuclei and green signal = CD31. Scale bar = 50µm. Images were taken using an IX micro widefield microscope and are representative of n=3.

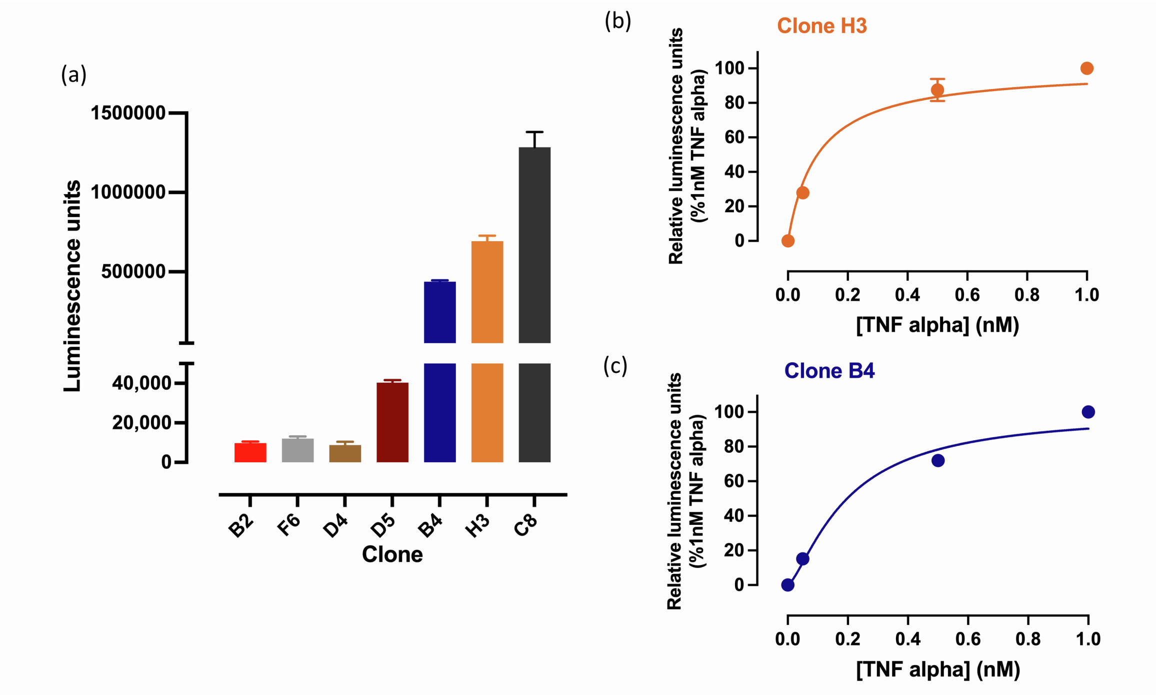

Figure S2. Screen of individual clonal lines isolated from CRISPR-Cas9 gene-edited TERT2 HUVECs. Related to Figure 3. (a) Raw luminescence for seven clonal TERT2-HUVEC cell lines stimulated with 1nM TNFα for 6h. (b,c) TNFα concentration-response curves for HiBiT-E-selectin expression in (b) H3 and (c) B4 cell lines. Values represent mean $\pm$ S.E.M of 3 independent experiments yielding EC_{50} values of 0.68 $\pm$ 0.13 nM (n=3) and 0.25 $\pm$ 0.01 nM (n=3) for clones B4 and H3 respectively.

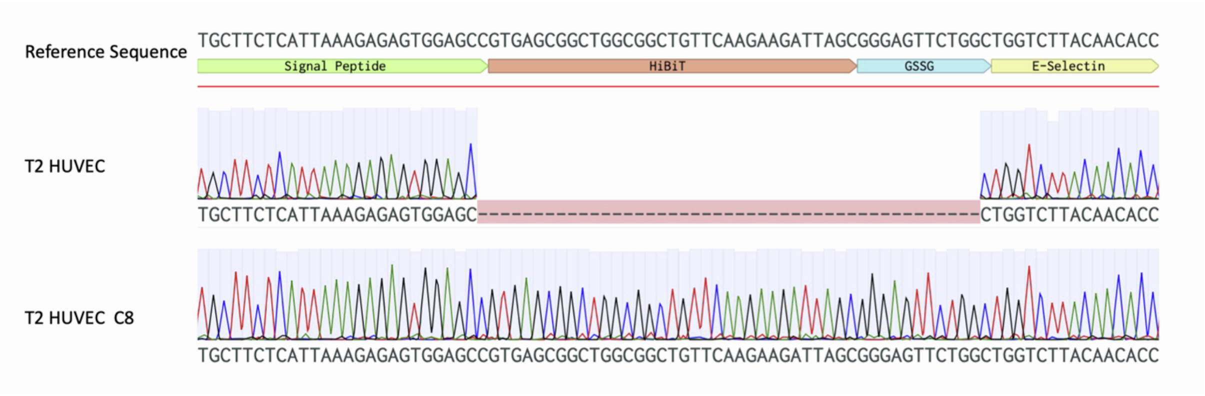

Figure S3. Nucleotide sequence of wild-type and clone C8 HiBiT-E-selectin DNA. Related to Figure 2. The sequence shows the presence of the HiBiT and GSSG linker only in the gene-edited HiBiT-E-selectin sequence of clone C8.

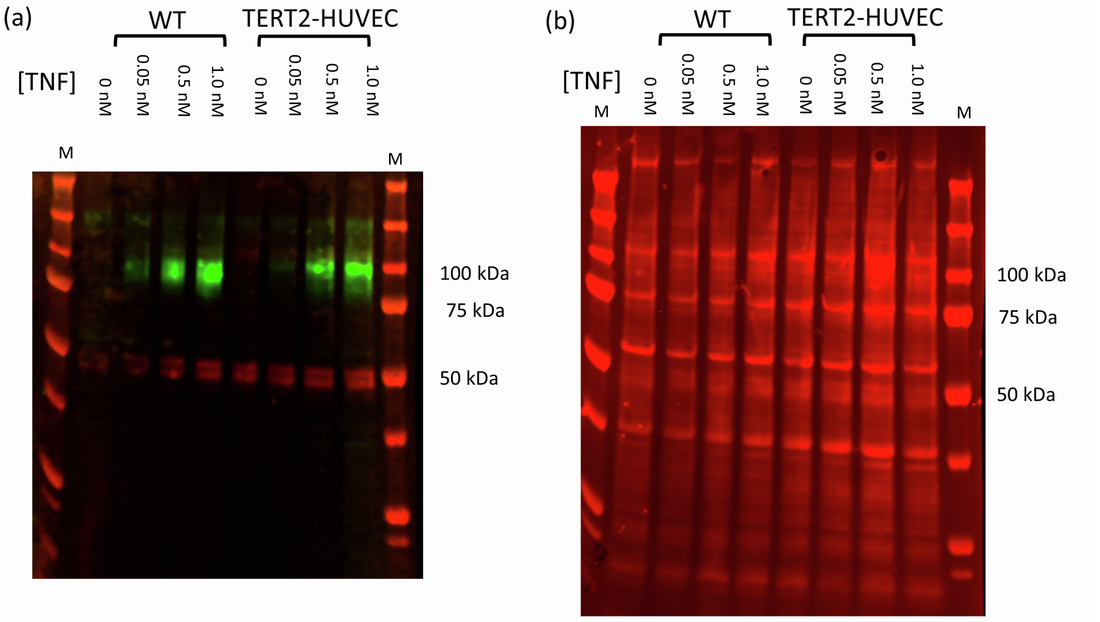

Figure S4. Western blot analysis of E-selectin expression in wildtype (WT) HUVECs and TERT2-HUVECs. Related to Figure 2. (a) Blot shows the effect of increasing concentrations of TNFα on E-selectin expression probed with an anti-E-selectin monoclonal antibody (green) or anti-alpha tubulin antibody (red). (b) The same blot as in (a) stained with Li-Cor Revert^{TM} 700 total protein stain to confim equal loading conditions.

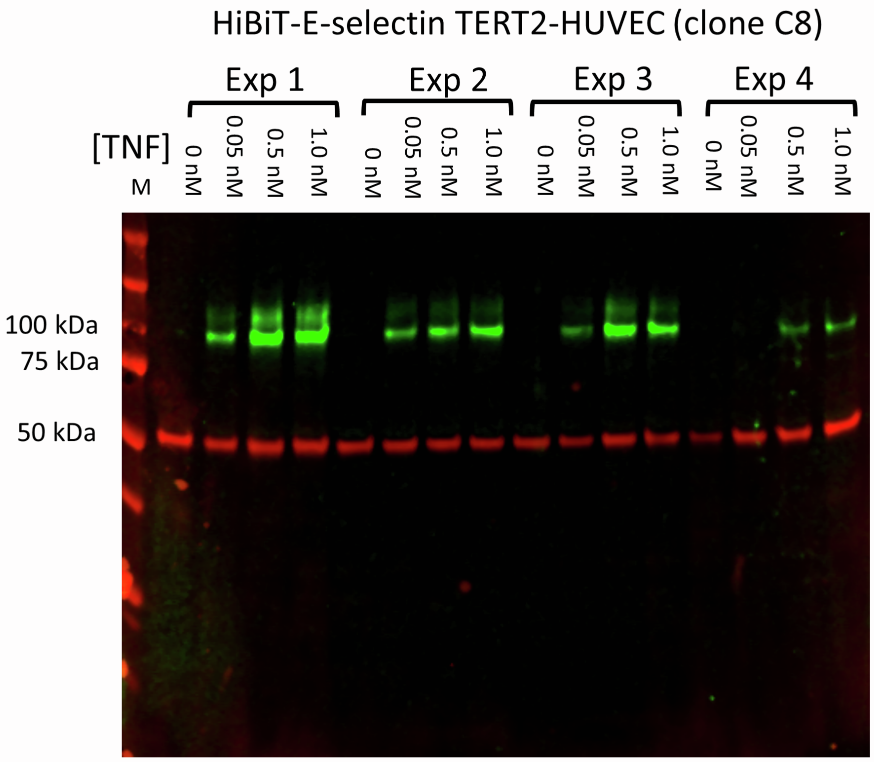

Figure S5. Western blot analysis of E-selectin expression in gene-edited HiBiT-E-selectin TERT2-HUVECs (clone C8). Related to Figure 2. Blots show the effect of increasing concentrations of TNFα on E-selectin expression. Data show blots from four independent experiments probed with an anti-E-selectin monoclonal antibody (green) or anti-alpha tubulin antibody (red).

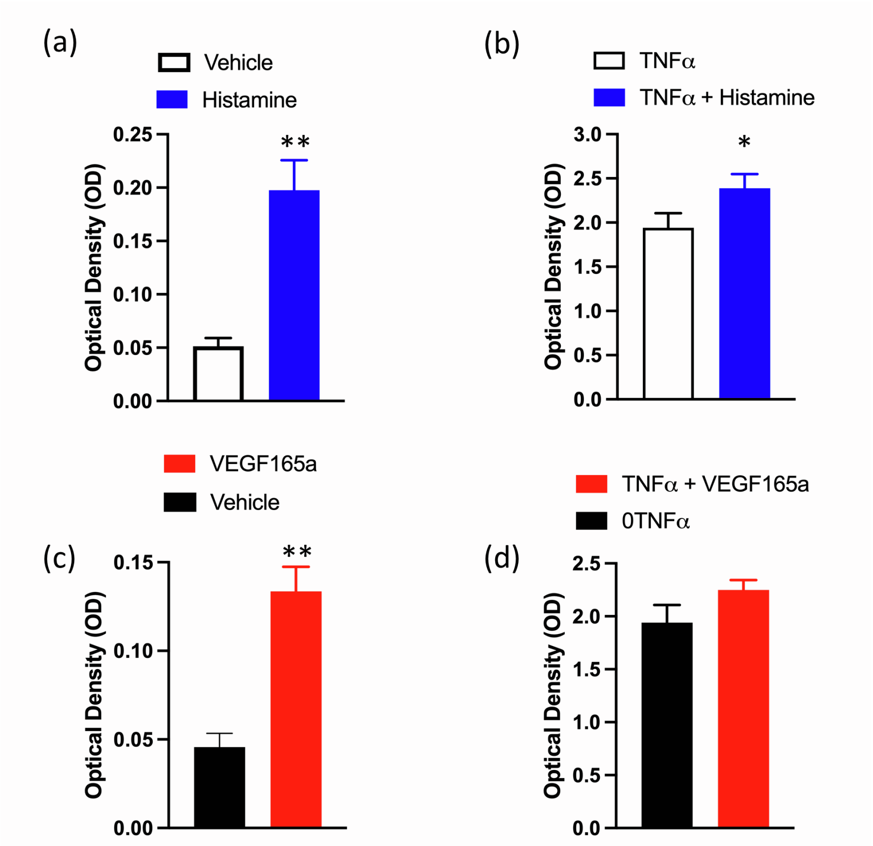

Figure S6. Effect of histamine and VEGF$_{165}$a on E-selectin expression in un-transfected HUVECs. Related to Figure 6. Quantification of E-selectin expression in WT-HUVECs using an alkaline phosphatase secondary antibody and p-nitrophenol phosphate (pNPP) substrate. Cells were stimulated with 100nM histamine, 100nM VEGF$_{165}$a or 1nM TNFα for 8h. Values show optical densities obtained in five separate experiments in response to (a) histamine alone, (b) a combination of 100nM histamine and 1nM TNFα, (c) VEGF$_{165}$a alone or (d) a combination of 100nM VEGF$_{165}$a and 1nM TNFα. Values are mean ± S.E.M. of five separate experiments each performed in triplicate. **$p<0.01$ compared to vehicle control (paired t test). * $p<0.05$ compared to TNFα alone (paired t test).
